# Case of Supposed Confluent Varicella

**Published:** 1815-04

**Authors:** Matth. Rowe

**Affiliations:** Member of the Roy. Coll. of Surg. 1, Clarendon-square, Somers'-town


*For the Medical and Physical Journal.*

### *Case of supposed Confluent Varicella;* by Mr. Rowe.

FROM the difference of opinion existing with medical men respecting vaccination being a preventative of variolous contagion, I am strongly induced to publish this case, so much resembling both varicella and small-pox.

On the 17th of February last, I was sent for to see a lad, a pupil of Mr. Firminger's, Camden-street, Camden-town; I found him labouring under a great deal of fever; pulse quick; tongue dry, and of a dark colour; skin very hot, and the face much flushed; a rash could easily be felt under the skin, which was not at all elevated; the eyes were a good deal inflamed, and the throat sore. From these symptoms, I was induced to believe it was either measles or scarlatina. I ordered him an aperient at bed-time, composed of calomel and rhubarb, which operated freely towards morning, when I found him much better; the fever abated, and the face very full of small pustules, a numerous quantity of which were scattered all over the body, the arms in particular; but these were not so much elevated as those on the face.

The whole bore a very strong resemblance to small-pox, but upon enquiry it was ascertained, that he had been vaccinated three or four years before, and that the disease had gone through its regular form. The following circumstance also tended very strongly to heighten my doubts as to the nature of the disease: six or seven boys of the school were then affected by varicella in its usual mild form; his brother, one of the number, with whom he slept, and who had also been vaccinated. On visiting him in the evening, I found no alteration in the symptoms.

19th. The pustules were more diffused and elevated; the eyes a little swollen; fever high, and pulse quick; but no dangerous symptoms present. Much the same in the evening.

20th.

20th. Still little variation of symptoms; the pustules larger, and filled with a lymph of a bright colour.

21st. I found the fever much abated, and my patient in every respect going on well. The treatment pursued throughout, had been simply that employed in all similar diseases—the bowels kept open with small doses of calomel and antimony, the former of which acted slightly on the salivary glands.

22d. The pustules had changed in appearance, and contained a straw-coloured lymph; and, on the 23d, three or four of those on the face were on the decline; others filled with *pus*.

24th. The eruption was still more declining on the face; but the pustules on the extremities continued full and large, and remarkably firm to the touch, feeling under the hand like pease.

25th. They were all very much changed in appearance, and the boy quite well.

I continued to see him daily till the 2d of March, when I found the pustules had not left a depression, as is usual in small-pox, but that the skin was universally elevated; this symptom induced me more strongly to believe in its being varicella.

In a case of so much interest, at the same time affording such ample room for variety of conjecture and difference of opinion, I felt unwilling to rely upon my own judgment alone, and therefore solicited the attention of several of my medical friends, some of whom saw my patient daily, and thus had an opportunity of observing the disorder in all its progressive and receding stages. Among them, he was visited by a physician, to whom the medical world is indebted for a most useful and interesting work on Cutaneous Diseases; his opinion was equally fluctuating and undecided, from the disorder bearing so strong a similarity to small-pox, while at the same time so many circumstances corroborated the supposition of its being varicella.

Some pus was taken for the purpose of inoculation, and I hope by the next month to have it in my power to give this demonstrative and decisive proof. I feel most anxious, that on this subject conjecture should be satisfied; for I have no doubt that many cases of the kind have occurred in families, where the medical attendant has never thought of varicella; thereby tending to prejudice him against, and depreciate the value of, that inestimable discovery (vaccination), to which we are indebted for the preservation of the lives of thousands.

1, *Clarendon-square, Somers'-town;* MATTH. ROWE.

*March* 3, 1815. *Member of the Roy. Coll. of Surg.*

